# Effect of microencapsulation on concentration of isoflavones during simulated in vitro digestion of isotonic drink

**DOI:** 10.1002/fsn3.929

**Published:** 2019-01-30

**Authors:** Dorota Wyspiańska, Alicja Z. Kucharska, Anna Sokół‐Łętowska, Joanna Kolniak‐Ostek

**Affiliations:** ^1^ Department of Fruit, Vegetable and Plant Nutraceutical Technology Wrocław University of Environmental and Life Sciences Wrocław Poland

**Keywords:** in vitro digestion, Isoflavones, isotonic drinks, microencapsulation

## Abstract

Isotonic drinks, fortified with microencapsulated maltodextrin and inulin isoflavone extract, were evaluated by organoleptic assessment and color measurement on the CIE L*a*b scale, then they were subjected to in vitro digestion. Changes of concentrations of isoflavones released during digestion of drinks and their ability to neutralize free radicals (ABTS) were determined. The effect of microencapsulation and storage on isoflavone content in isotonic drinks was also evaluated. In the organoleptic evaluation, beverages without additives and beverages fortified with microencapsulated extract were evaluated as better than a beverage with pure extract. Microencapsulation largely eliminated the unpleasant taste and odor of isoflavones and masked their color. Before digestion, isotonic drinks contained 29.07–43.17 μg of isoflavones in 1 ml. Microencapsulated isoflavones were released gradually during simulated digestion. The highest recovery of these compounds was observed for glycoside and malonylate derivatives of daidzein. Daidzin and genistin with encapsulation showed bioavailability of 0.02%–0.07%. The use of inulin as a carrier increased the stability of microcapsules during the storage of isotonic beverages. Microencapsulated of isoflavones from soybeans can increase the health benefits of conventional beverages available on the market.

## 1 INTRODUCTION

Isotonic drinks are an example of functional foods. They are designed for athletes, people with weakened immunity, and those who are intensively working. They contain a set of easily digestible carbohydrates and appropriately balanced composition of minerals. These drinks enable quick replenishment of electrolytes lost in sweat and rapid correction of water loss in the body. They also contribute to maintenance of normal blood glucose levels (Vera de Rosso & Mercadante, 2007).

In recent years, there has been growing interest in isotonic drinks, which are taking an increased share of the soft drinks market. Greater competition in the food market has led to the introduction of new, improved products. Increasingly, manufacturers supplement drinks with antioxidants, to protect the muscles against excessive free radicals (Joachimiak & Szołtysek, 2013). However, not all active ingredients isolated from plants are of attractive flavor and stable during storage. Isoflavones are an example of such compounds—bioactive, but unappealing in taste.

Isoflavones belong to the group of phytoestrogens. Their construction is similar to the female sex hormone 17‐β‐estradiol. They exhibit antioxidant and antiestrogenic activity and act as inhibitors of protein tyrosine kinase (Czerpak, Pietryczuk, Jabłońska‐Trypuć, & Orębska, 2009). Genistein and daidzein inhibit the growth of breast cancer cells and hepatic cancer cells (Kim, Kim, Hahn, & Chung, 2005). Epidemiological studies show that isoflavones may be of importance as dietary supplements in hormone replacement therapy, mitigating the symptoms of menopause, in chemoprevention of cancer and in the prevention of cardiovascular disease (Dixon & Ferreira, 2002). However, the extract isolated from soybeans has a bitter and unpleasant taste (Kim, Jeon, Ahn, & Kwak, 2006). A chance to achieve attractive composition of the beverage with isoflavones from soy, having a taste acceptable for consumers and with increased stability, is the process of microencapsulation, which uses various media.

Encapsulation involves the closure of a solid, liquid, or gas in a capsule, which may release its contents in a controlled manner under appropriate conditions (Desai & Park, 2005). The most common method to increase the stability of the active compounds is microencapsulation using spray drying. Typical materials used as carriers in spray drying are maltodextrin, inulin, and modified starch (Bąkowska‐Barczak & Kołodziejczyk, 2011). Research shows that microencapsulation can also extend the half‐life of the active compounds in the body (Setchell et al., 2005). Half‐life is linked to the concept of bioavailability, which determines the degree to which a nutrient is absorbed by the body. The bioavailability of the different nutrients is influenced both by the type of feed matrix and the degree of interaction between the various components of food (Parada & Aguilera, 2007). Bioavailability is a function of a given component matrix susceptibility to intestinal digestion and diffusion properties. Due to difficulty in accessing the intestinal contents to assess the stability of the various components during the digestion process, in vitro simulated digestion was developed.

In recent years, there has been considerable progress in the study of the stability of bioactive compounds under simulated in vitro conditions. Seok, Kim, and Kwak (2003) investigated the sensory properties of microcapsules of isoflavones added to milk and their stability during simulated in vitro digestion. Walsh, Zhang, Vodovotz, Schwartz, and Failla (2003) determined the effect of simulated digestion on the stability and bioavailability of soy bread isoflavones. In their studies, Kim et al. (2006) determined the optimal conditions of isoflavone microencapsulation; the coating material was medium‐chain triacylglycerol (MCT) or polyglycerol monostearate (PGMS). The authors determined the effectiveness of the release of the microcapsules in simulated conditions of the gastrointestinal tract. However, up to now, stability studies of isoflavones in the form of microcapsules added to isotonic drinks during their in vivo digestion have not been carried out. Therefore, the aim of this study was to determine the effect of microencapsulation using maltodextrin and inulin on the stability and antioxidant activity of isoflavones during simulated in vitro gastrointestinal digestion of isotonic drinks enriched with these components. Also, the impact of the microencapsulation process on color according to the CIE L*a*b* scale and overall acceptability of the isotonic drinks was evaluated.

## 2 MATERIALS AND METHODS

### 2.1 Chemicals

The compounds 2,2′‐azinobis‐(3‐ethylbenzothiazoline‐6‐sulfonic acid) (ABTS), 6‐hydroxy‐2,5,7,8‐tetramethylchroman‐2‐carboxylic acid (Trolox), acetonitrile, formic acid, pepsin (3,200–4,500 units/mg protein), pancreatin (8× USP), and bile salts were obtained from Sigma‐Aldrich (Steinheim, Germany). Acetone, NaHSO_3_, anhydrous glucose, sodium benzoate, sodium citrate, and potassium phosphate were purchased from Chempur (Piekary Slaskie, Poland). Acetonitrile LC‐MS came from POCh (Gliwice, Poland), fructose from Biofan (Piekary Slaskie, Poland), sodium chloride and potassium chloride from STANLAB (Lublin, Poland). Daidzin and genistin were purchased from Extrasynthese (Genay, France).

### 2.2 Obtaining the extract of isoflavones from soybeans

The methodology was developed according to the procedure described by Wanezaki and Araki (2009).

The milled soya beans were treated with hexane for degreasing. Then a double extraction with 80% ethanol was performed. The samples after extraction were sonicated (15 min) and centrifuged. The obtained extracts were concentrated at 40°C under reduced pressure. The extract was applied to a column filled with Amberlite XAD 16 resin. Isoflavones were eluted with 80% ethanol. The ethanolic solution obtained was concentrated in a vacuum and then dried (39–40°C, 0.094 MPa). The preparation yield was 0.8%.

### 2.3 Spray drying

Spray drying was performed according to Wyspiańska, Kucharska, Sokół‐Łętowska, and Kolniak‐Ostek (2017). Inulin Orafti HPX (DP ≥ 23) was bought from HORTIMEX PLUS (Konin, Poland). Maltodextrin (8 DE) came from the Department of Food Storage and Technology of Wroclaw University of Environmental and Life Sciences. Isoflavones from soybean were suspended in water to give a 10% w/v dispersion (Wanezaki & Araki, 2009). To the solution, in the appropriate proportions, inulin and maltodextrin were added, so that the ratio of product to medium was 1:5 (isoflavones:carrier). The solutions were homogenized (2 min) and spray dried in a mini spray dryer (BUCHI, Flawil, Switzerland). The inlet air temperature was 150°C and the raw material feed flow: 615 ml/hr. Temperature of the raw material was 40°C.

### 2.4 Isotonic drinks design

Isotonic drinks were prepared according to a formula developed by Vera de Rosso and Mercadante (2007). One liter of beverage contained the following constituents: 55 g of sucrose, 5.5 g of fructose, 5.5 g of glucose anhydrate, 0.15 g of sodium benzoate as conservants, 0.14 g of sodium citrate, 0.5 g of sodium chloride, 0.5 g of potassium chloride, 0.4 g of potassium phosphate, 50 mg of extract of soybeans isoflavones, or 250 mg of the microcapsules with inulin or maltodextrin (ratio of extract to the carrier was 1:5). Beverages were pasteurized at 80°C for 30 s. Samples were labeled as follows: IC: control sample; IE: isotonic drinks with pure extract of isoflavones; II: isotonic drinks with isoflavones encapsulated with inulin; IM: isotonic drinks with isoflavones encapsulated with maltodextrin. The resulting drinks were stored at 4°C and 30° for 12 weeks.

### 2.5 Scanning electron microscopy (SEM) of microcapsule powder

Morphology and size of the microcapsule powder were evaluated by scanning electron microscopy (SEM). The *Zeiss* Supra 35 (Berlin, Germany) electron microscope was equipped with an electron gun with field emission, a GEMINI electron optical column, and an oil‐free vacuum system. A layer of gold coating (2 nm) was applied to each of the mounted samples prior to analysis. Observations were made at magnifications in the range of 5 000–25 000.

### 2.6 Efficiency of microencapsulation

The efficiency of microencapsulation was evaluated on the basis of the method developed by Zhang et al. The efficiency was calculated by the following formula:

$$1-\frac{m_{\mathrm{surf}}}{m_{\mathrm{tot}}}$$

where *m*_surf_ is the amount of isoflavones on the microcapsule surface and *m*_tot_ is the total amount of isoflavones in the products.

### 2.7 Identification of isoflavones by LC‐MS

The LC‐MS analysis of isoflavones was performed as described previously by Wyspiańska et al. (2017). Isoflavones identification was performed on an Acquity ultraperformance liquid chromatography (UPLC) system, coupled with a quadrupole time‐of‐flight (Q‐TOF) MS instrument (UPLC/Synapt Q‐TOF MS; Waters Corp., Milford, MA, USA), with an electrospray ionization (ESI) source. Separation was achieved on the Acquity TM BEH C18 column (100 × 2.1 mm i.d., 1.7 μm; Waters). The mobile phase was a mixture of 4.5% v/v aq. formic acid (A) and acetonitrile (B). The gradient program was as follows: initial conditions, 1% B; 12 min, 25% B; 12.5 min, 100% B; 13.5 min, 1% B (return to initial conditions). The flow rate was 0.45 ml/min, and the injection volume was 5 ml. The column was operated at 30°C. The major operating parameters for the Q‐TOF MS were consistent with the description of Wyspiańska et al. (2017). The run was monitored at 254 nm.

### 2.8 Quantitative determination of isoflavones by HPLC‐DAD

The HPLC‐DAD analysis of isoflavones was performed as described previously by Wyspiańska et al. (2017). The HPLC analysis was performed using a Dionex (Germering, Germany) system. A Cadenza Imtakt column C18 (75 × 4.6 mm) was used. The mobile phase was composed of solvent A (4.5% formic acid, v/v) and solvent B (100% acetonitrile). The elution system was as follows: 0–1 min 5% B, 20 min 25% B, 26 min 100% B, 27 min 5% B, 30 min: 5% B. The flow rate of the mobile phase was 1.0 ml/min and the injection volume was 20 μl. The column was operated at 30°C. Isoflavones were detected at 280 nm. Isoflavones were quantified as daidzin and genistin. All determinations were done in triplicate (*n* = 3) and the results were expressed as μg per ml.

### 2.9 Sensory evaluation

The evaluation of isotonic drinks was performed using the scalar method on a five‐point scale according to the standard ISO 4121 (1998), where one corresponds to the lowest, and five to the highest rating. Typical beverage quality factors were evaluated: clarity, color, aroma, and flavor. Sensory assessment was performed by a team consisting of ten trained staff with the necessary sensitivity to sensory impairments, who used previously developed ratings tables.

### 2.10 Colorimetric analysis

Color measurements were made using the L*a*b* instrumental scale, using a Color Quest XE colorimeter (HunterLab, Reston, VA, USA) in transmitted light, 10° observer and illuminant D65.

### 2.11 In vitro gastrointestinal digestion

Simulated digestion was performed according to the methodology described by Gawlik‐Dziki (2007) with modifications.

#### 2.11.1 Gastric digestion

In the first step, isotonic drinks (10 ml) were placed in a beaker and adjusted to pH 1.2 by addition of an appropriate amount of 6 M HCl. Then, 30 ml of gastric fluid (pepsin, Sigma product number P6887‐1G) 0.32% solution in 0.03 M NaCl, pH 1.2 was added to the samples. The mixtures were incubated for 2 hr at 37°C in a shaking water bath and protected from light. At the end of this stage, samples were collected for further analysis, and the residue was subjected to intestinal digestion.

#### 2.11.2 Intestinal digestion with dialysis membrane

In the next step, each sample was adjusted to pH 5.0 using 0.5 M NaHCO_3_, and the amount of 0.5 M NaHCO_3_ needed to be added to the sample to adjust the pH to 7.5 was calculated. The dialysis membrane (D9777‐100FT 25 mm) was filled with the calculated amount of 0.5 M NaHCO_3_. After creating the membranes, they were placed in a beaker, for immersion in the solution, then they were submitted to a water bath at 37°C with shaking for 30 min. After 30 min, 30 ml of mixture prepared from 0.05 g of pancreatin and 0.3 g of bile extract (Sigma product number P7545 and B8756‐10G`1) and dissolved in 35 ml 0.1 M NaHCO_3_ was added to each beaker, and then placed in a shaking water bath for 90 min. After incubation at 37°C in a water bath, a solution of the beakers from the outside of the membrane and inside the dialysis tube was sampled for analysis. Tests were performed in triplicate. At all stages of digestion, qualitative and quantitative changes of the active compounds were determined by LC‐MS and HPLC. For this purpose, the samples were centrifuged (4 °C, 21,600 x *g* for 8 min), diluted, and purified on Whatman filters (0.45 μm—HPLC; 0.22 μm—LC‐MS). Before performing the determinations of antioxidant activity, the sample was purified on Sep‐Pak C18 minicolumns.

### 2.12 Bioavailability calculations

Bioavailability indicates the fraction of the nutrient or bioactive compound ingested that is available for use in physiologic functions or to be stored (McDougall, Dobson, Smith, Blake, & Stewart, 2005).

$$\text{BIOAVAILABILITY}\;\left[\%\right]=\frac{A}{B}\times 100\%$$

*A*, amount of active compounds in a given stage of digestion (μg)

*B*, amount of active compounds before the process of digestion (μg).

### 2.13 Antioxidant capacity

All determinations were performed in triplicate using a Shimadzu UV‐2401 PC spectrophotometer (Kyoto, Japan). The ABTS activity of samples was determined according to the method of Re et al. (1999). For all analyses, the standard curve was prepared using different concentrations of Trolox. The results were corrected for dilution and expressed in nmol Trolox equivalent (TE) per ml.

### 2.14 Statistical analysis

The results were analyzed statistically by Statistica 10.0 software, using one‐way analysis of variance (ANOVA). Differences were rated by Duncan's test at the significance level α = 0.05.

## 3 RESULTS AND DISCUSSION

### 3.1 Morphology and particle size of microencapsulated isoflavones (isoflavone microcapsules)

**Figure 1:**
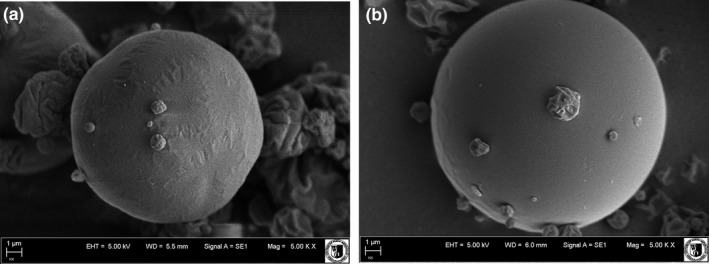
SEM and microencapsulation efficiency of the isoflavone extract from soybeans, encapsulated with maltodextrin (a) and inulin (b)

SEM images of the isoflavone extract from soybeans encapsulated with maltodextrin and inulin are illustrated in Figure 1. During the spray drying of the soy extract with the carriers, molecules differing in morphological structure were created. Both spherical particles with a smooth undamaged surface as well as smaller and irregular particles can be observed. The use of maltodextrin during spray drying was associated with the formation of particles of various sizes, with a slightly wrinkled surface or with deep depressions. A better surface structure of the capsules was obtained using inulin as a carrier. This result may suggest that the product will have better storage stability. According to Kanakdande, Bhosale, and Singhal (2007), microcapsules should have a homogeneous and smooth surface, with a slightly spherical shape with minimal cracks and wall creases. Microcapsules with rough surfaces are more sensitive to the oxidation reaction compared to smooth surfaces. The resulting microcapsules had a diameter of 2 (small and irregular) to 10 μm (big and regular without pinhole and dents). The largest ones did not have any indentation, which suggests that they will be less exposed to external factors. A slightly larger diameter of the capsules was obtained by Barros Fernandes, Borges, and Botrel (2014)for microcapsules made using starch (13.4 μm) and arabic gum (13.5 μm). The diameter of the spray dried particles depends on the properties of the coating material, the concentration and viscosity of the encapsulated material, and the drying conditions (Jafari, Assadpoor, He, & Bhandari, 2008).

### 3.2 Efficiency of microencapsulation

The efficiency of microcapsulation is shown in Figure 1. The use of inulin as a carrier during spray drying was associated with a smaller amount of isoflavones marked on the surface of the microcapsule compared to its center, and thus better efficiency (64%). In the case of microcapsules coated with maltodextrin, the efficiency was 48%.

The lower the efficiency of the process, the greater the losses of compounds during storage. The effectiveness of encapsulation depends on various factors, such as the concentration of the polymer and its solubility as well as the rate of solvent evaporation during spray drying (Venkata Naga Jyothi et al., 2010). In the studies of Mehta, Thanoo, and DeLuca (1996), the efficiency of encapsulation increased from 53.1% to 70.9% when the polymer concentration increased from 20.0% to 32.5%.

According to Janiszewska, Cupiał, and Witrowa‐Rajchert (2008), the efficiency of microcapsulation depends on the used carrier, method, and temperature prevailing in the spray dryer. Dłużewska, Florowska, and Jasiorska (2011) showed that modified starch microcapsules had a higher concentration of β‐carotene on the surface than arabic gum microcapsules, regardless of the amount of starch added.

### 3.3 Sensory evaluation of isotonic drinks

In the natural sequence of sensory evaluation of a product's first appearance, color and clarity, and next flavor and taste are taken into account. The color should be attractive and eye‐catching, while the aroma and flavor should be pleasant and encouraging for consumption. Results of sensory evaluation of isotonic drinks without (IC) and with the addition of isoflavones from soy (IE, IM, II) on a scale of 1 to 5 points are given in Table 1. The beverage without additives in the evaluation of the color and clarity was rated high, that is, 4.6 and 5.0 points, respectively (on a scale of 1 to 5 points), and the aroma and taste rather low, that is, 3.6 and 4.1 points, respectively. The addition of an extract of soy adversely affected the color and clarity of the beverage. Drinks were evaluated from 1 to 1.6 points lower than the control sample. Addition of isoflavone capsules to beverages was evaluated better. The color of beverages with capsules was judged to be comparable to the control (4.6 points). In the evaluation of clarity, beverages IM (4.7 points) and II (4.1 points) have a higher rating as compared to the sample with pure extract. According to the aroma and taste of beverages, a higher rating was obtained by samples M and II than the control sample and the sample with an extract of soybeans. Beverage IE was characterized by a bitter aftertaste of isoflavones, with a beany off‐flavor—it was the cause of its low score (3.7 points). The process of microencapsulation made it possible to overcome the unpleasant taste and odor of isoflavones; hence, evaluation of beverages IM and II was higher or comparable to the control. A similar effect was obtained by other authors studying addition of isoflavone capsules to milk. Byung‐Ju, Nam‐Chul, Eun‐Mi, and Hae‐Soo (2005) reported that bitterness, astringency, and off‐taste in the encapsulated isoflavone‐added milk were slightly, but not significantly, different from those in noncapsulated, isoflavone‐added milk.

**Table 1:** Sensory evaluation of isotonic drinks

Sample	Color	Clarity	Aroma	Flavor	Sum
IC	4.62 ± 0.11^a^	5.03 ± 0.12^a^	3.61 ± 0.12^b^	4.13 ± 0.21^b^	7.21 ± 0.11^b^
IE	3.65 ± 0.13^b^	3.45 ± 0.14^d^	4.03 ± 0.11^a^	3.72 ± 0.12^c^	6.14 ± 0.12^c^
IM	4.61 ± 0.21^a^	4.77 ± 0.35^b^	3.96 ± 0.22^a^	4.75 ± 0.11^a^	7.43 ± 0.11^a^
II	4.63 ± 0.14^a^	4.19 ± 0.23^c^	4.09 ± 0.13^a^	4.17 ± 0.14^b^	7.01 ± 0.11^b^

Data are expressed as mean ± SD; (*n *=* *10).

IC: control sample; IE: isotonic drinks with pure extract of isoflavones; II: isotonic drinks with isoflavones encapsulated with inulin; IM: isotonic drinks with isoflavones encapsulated with maltodextrin.

Superscript letters “a,b,c” statistically homogeneous groups within the same column (Duncan's test *p *≤* *0.05).

### 3.4 Color of isotonic drinks

Isotonic drinks’ color measurement was performed using the CIE L*a*b system. The results are shown in Table 2. In isotonic drinks, color plays an important role, being a significant differentiator in consumer evaluation. To improve the attractiveness of this product group, commercially available artificial colors (Brilliant Blue—E133, Sunset Yellow—E110) are added to drink (Gironés‐Vilaplana, Villaño, Diego, Moreno, & Garcia‐Viguera, 2013). The novel isotonic drinks enriched with microcapsules designed in this study were characterized by an attractive bright color. It seems that this enrichment can be an alternative to artificial colorants.

**Table 2:** Color parameters (L*, a*, b*) of isotonic drinks

Sample	L*	a*	b*
IC	86.62 ± 0.11^a^	−0.02 ± 0.11^b^	1.43 ± 0.11^d^
IE	68.77 ± 0.12^d^	0.31 ± 0.13^a^	7.56 ± 0.21^a^
IM	81.85 ± 0.23^c^	−0.23 ± 0.12^c^	2.34 ± 0.34^b^
II	84.02 ± 0.14^b^	−0.28 ± 0.11^d^	1.90 ± 0.12^c^

Data are expressed as mean ± SD; (*n *=* *3).

IC: control sample; IE, isotonic drinks with pure extract of isoflavones; II, isotonic drinks with isoflavones encapsulated with inulin; IM: isotonic drinks with isoflavones encapsulated with maltodextrin.

Superscript letters “a,b,c” statistically homogeneous groups within the same column (Duncan's test. *p *≤* *0.05).

The test beverages significantly differ from each other in the value of the L* parameter, which determines the brightness. The L* value for the control beverage without additives was 86.62 units. All additions caused darkening of drinks, but drinks containing microcapsules were clearly brighter than beverages with the addition of extract. The L* value for IM and II was, respectively, 81.85 and 84.02, while for IE the value was 68.77 units. The addition of microcapsules coated with inulin makes it possible to maintain a light‐colored beverage, closest to the control sample. The a* parameter values for drinks containing microcapsules and the control beverage were similar and had a negative range (from −0.28 [II] to −0.02 [C]), while for the beverage with the extract the parameter had a positive value (0.31). The b* parameter, which indicates the location in the color space between the colors yellow and blue, for the control sample without the additive was 1.43 units, while for drinks with additives IE, IM, and II were, respectively, 7.56, 2.34, and 1.90. The microencapsulation process made it possible to mask the color of isoflavones in isotonic drinks and reduce the share of yellow color. In a study of microencapsulated polyphenols in cherry, Cilek, Luca, Hasirci, Sahin, and Sumnu (2012) demonstrated that the process of microencapsulation caused brightening of the extract and reduction of the proportion of red color.

### 3.5 Qualitative analysis of isoflavones

Results of identification of compounds contained in isotonic drinks are shown in Table 3, Supporting Information Figures S1 and S2. The structural characteristics of the compounds of the soybean were based on retention times, UV‐Vis and mass spectra and fragmentation ions. The results were compared to available standards and literature data (Chen, Zhao, Plummer, Tang, & Games, 2005; Klejdus et al., 2005). The compounds included in the beverages were characterized by typical UV spectra of isoflavones with maximum absorbance at 260 nm. Among these compounds, three glycosidic derivatives, five esters with malonylic acid, and two aglycones were identified. Compounds 1–3 were characterized as daidzin (*m/z* 417.1021; *t*_R_ = 6.01 min), glycitin (*m/z* 447.1432; *t*_R_ = 6.48), and genistin (*m/z* 433.1172; *t*_R_ = 7.53). After fragmentation ions with [M+H]^+^ at *m/z* 255.1432, *m/z* 285.1324 and *m/z* 271.1032 corresponding to daidzein, glycitein, and genistein aglycones were obtained. Ions connected to the aglycones were a simple sugar—hexose (162 Da). Among malonylic derivatives (compounds 4–8), two isomers of 6″‐*O*‐malonyldaidzin (*m/z* 503.1042; *t*_R_ = 7.65 min and 8.46 min), 6″‐*O*‐malonylglycitin (*m/z* 533.1413; *t*_R_ = 8.74 min) and two isomers of 6″‐*O*‐malonylgenistin (*m/z* 519.1181; *t*_R_ = 9.23 min and 9.92 min) were identified. After fragmentation, ions with [M+H]^+^ at *m/z* 255.1432, *m/z* 285.1324, and *m/z* 271.1032 characteristic for daidzein, glycitein, and genistein aglycones, respectively, were identified. Fragmentation patterns indicate the attachment of one molecule of hexose (162 Da), and one malonylic group—[‐COCH_2_COOH] (86 Da). Compounds 9 and 10 had molecular ions at *m/z* 255.1432 (*t*_R_ = 10.39 min) and 271.1032 (*t*_R_ = 12.60 min), which corresponded to the molecular ions of aglycone daidzein and genistein, respectively. This profile is typical for soy isoflavones, from which the extract was prepared, and is in accordance with literature data (Konar, Poyrazo, & Demir, 2012).

**Table 3:** Qualitative characteristics of phenolic compounds in isotonic drinks by LC‐MS

Peak no.	*t*_R_ (min)	λ_max_ (nm)	[M+H]^+^ (*m/z*)	MS/MS (*m/z*)	Neutral loss (De)	Proposed formula	Exact mass (*m/z*)	Measured mass (*m/z*)	Δm (ppm)	Tentative identification
1	6.01	254	417.1021	255.1432	162	C_21_H_20_O_9_	416.378	416.094	0.28	Daidzin
2	6.48	254	447.1432	285.1324	162	C_22_H_22_O_10_	446.404	446.135	0.27	Glycitin
3	7.53	254	433.1172	271.1032	162	C_21_H_20_O_10_	432.377	432.109	0.27	Genistin
4	8.22	254	503.1042	255.1432	248/(86 + 162)	C_24_H_22_O_12_	502.424	502.096	0.33	6″‐*O*‐malonyldaidzin
5	8.46	254	503.1042	255.1432	248/(86 + 162)	C_24_H_22_O_12_	502.424	502.096	0.33	6″‐*O*‐malonyldaidzin
6	8.74	254	533.1413	285.1324	248/(86 + 162)	C_25_H_24_O_13_	532.450	532.133	0.32	6″‐*O*‐malonylglycitin
7	9.23	254	519.1181	271.1032	248/(86 + 162)	C_24_H_22_O_13_	518.424	518.110	0.31	6″‐*O*‐malonylgenistin
8	9.92	254	519.1181	271.1032	248/(86 + 162)	C_24_H_22_O_13_	518.424	518.110	0.31	6″‐*O*‐malonylgenistin
9	10.39	254	255.1432	–	–	C_15_H_10_O_4_	254.238	254.094	0.14	Daidzein
10	12.60	254	271.1032	–	–	C_15_H_10_O_5_	270.237	270.099	0.14	Genistein

### 3.6 Quantitative analysis of microencapsulated isoflavones

The results of the quantitative analysis of the microencapsulated isoflavones contained in isotonic drinks are shown in Table 4. Using maltodextrin, in isoflavones microencapsulation made it possible to obtain beverages richer in isoflavones, compared to inulin. In the beverages enriched with the spray dried extract with inulin and maltodextrin, isoflavones were identified at the levels 29.094 and 43.166 μg/ml, respectively. Higher polyphenol content in powder dried with maltodextrin than in powder dried with inulin was also observed by other authors (Bąkowska‐Barczak & Kołodziejczyk, 2011; Wyspiańska et al., 2017).

**Table 4:** Content of isoflavones (μg/ml) indicated by HPLC in IM and II beverages before and after each stage of digestion and antioxidant activity (ABTS) of isotonic drinks after simulated in vitro digestion (nmol TE/ml)

Sample	Compound (μg/ml)									TOTAL	ABTS (nmol TE/ml)
	Din	Glyn	Gin	MDin	MGlyn	MGin 1	MGin 2	Da	Ge		
Isotonic drinks with isoflavones encapsulated with maltodextrin (IM)											
IM_0_	4.253 ± 0.12^c^	0.642 ± 0.21 ^g^	27.271 ± 0.09^a^	2.762 ± 0.06^d^	0.351 ± 0.02^i^	0.732 ± 0.01^f^	5.432 ± 0.06^b^	1.292 ± 0.06^e^	0.431 ± 0.09 ^h^	43.166	570.84 ± 0.07^a^
IM_g_	0.112 ± 0.03^b^	–	0.294 ± 0.02^a^	0.103 ± 0.03^c^	–	–	0.121 ± 0.03^b^	0.042 ± 0.01^d^	0.044 ± 0.01^d^	0.716	300.99 ± 0.04^d^
IM_i_	0.313 ± 0.01^b^	–	0.742 ± 0.03^a^	0.191 ± 0.09^d^	–	–	0.211 ± 0.02^c^	–	–	1.457	90.54 ± 0.01^e,f^
IM_m_	0.003 ± 0.00^b^	–	0.006 ± 0.00^a^	–	–	–	–	–	–	0.009	40.12 ± 0.07^e,f,g^
Bioavailability %	0.07^a^	–	0.02^b^	–	–	–	–	–	–	0.09	
Isotonic drinks with isoflavones encapsulated with inulin (II)											
II_0_	2.863 ± 0.01^c^	0.294 ± 0.02^f^	19.151 ± 0.08^a^	1.884 ± 0.06^d^	0.232 ± 0.02 ^h^	0.191 ± 0.14^i^	3.313 ± 0.09^b^	0.894 ± 0.01^e^	0.272 ± 0.01 ^g^	29.094	320.10 ± 0.02^c^
II_g_	0.261 ± 0.03^d^	0.032 ± 0.01 ^g^	0.503 ± 0.09^a^	0.332 ± 0.08^c^	–	–	0.422 ± 0.07^b^	0.091 ± 0.01^f^	0.152 ± 0.01^e^	1.793	100.01 ± 0.01^e^
II_i_	0.252 ± 0.03^b^	–	0.532 ± 0.09^a^	0.133 ± 0.02^d^	–	–	0.171 ± 0.02^c^	–	–	1.088	80.94 ± 0.04^e,f,g^
II_m_	0.001 ± 0.00^b^	–	0.004 ± 0.00^a^	–	–	–	–	–	–	0.005	30.97 ± 0.06^e,f,g^
Bioavailability %	0.03^a^	–	0.02^a^	–	–	–	–	–	–	0.05	

Din, daidzin; Glyn, glycitin; Gin, genistin; MDin, 6″‐O‐malonyldaidzin; MGlyn, 6″‐O‐malonylglycitin; MGin, 6″‐O‐malonylgenistin; Da, daidzein; Ge, genistein.

Data are expressed as mean ± SD; (*n *=* *3).

Superscript letters “a,b,c” statistically homogeneous groups (Duncan's test. *p *≤* *0.05).

II: isotonic drinks with isoflavones encapsulated with inulin; IM: isotonic drinks with isoflavones encapsulated with maltodextrin; 0: before in vitro digestion, g: after gastric digestion, I: after intestinal digestion, m:after absorption through the membrane.

Among isoflavones identified in isotonic drinks with encapsulated extracts, three β‐glycosides, four malonylic derivatives, and two aglycone forms were quantified (for both IM0 and II0 samples). Genistin was the main isoflavone. In the beverages containing encapsulated extracts, genistin was about six times more abundant than daidzin, about five times more than 6″‐*O*‐malonylgenistin, and ten times more than 6″‐*O*‐malonyldaidzin. Lee et al. (2005) demonstrated that soybean is richest in 6″‐*O*‐malonylgenistin, followed by 6″‐*O*‐malonyldaidzin, genistin, and daidzin.

Aglycone forms of genistein and daidzein are present in the smallest amounts. Differences in comparison to literature data, concerning the malonylic and β‐glycoside compounds in the beverages with capsules may be the result of elevated temperatures used during the spray drying of the microcapsules and conversion of malonylic derivatives to the corresponding β‐glycosides. According to Chien, Hsieh, Kao, and Chen (2005), isoflavones are quite stable at temperatures up to 260°C. They are not degraded, but they undergo transformations between different forms. The mentioned authors studying the kinetics of degradation and conversion of the isoflavones at 100, 150, and 200°C noted conversion of 6″‐*O*‐malonylgenistin to genistein, 6″‐*O*‐malonylgenistin to 6″‐*O*‐acetylgenistin, and 6″‐*O*‐acetylgenistin to genistin and then genistein. According to Mathias, Ismail, Corvalan, and Hayes (2006), malonylic and acetyl derivatives are most stable at pH 2 and 7, and the transition occurs at pH 10 and at a temperature of 80–100°C. Regardless of pH, stability of malonylic and acetyl forms decreases with increasing temperature.

### 3.7 Simulated in vitro digestion of isotonic drinks

Table 4 shows the content of isoflavones indicated by HPLC in IM and II beverages before and after each stage of digestion. Already after the first stage of the simulated digestion, a significant decrease in the amount of isoflavones in all samples was observed. A similar reduction of isoflavones after the stage of simulated gastric digestion was observed by Piskula (2000). The author explained this as a lack of solubility of isoflavones in acidic conditions. Opposite observations were made by Rodríguez‐Roque, Rojas‐Graü, Elez‐Martínez, and Martín‐Belloso (2013), who found that after simulated gastric digestion, the concentration of isoflavones increased by 22% compared to their content in a nondigestible soy milk, by improving the release of the isoflavones from the food matrix by the action of the acid environment of the stomach. A similar hypothesis was discussed by other authors. Peńalvo, Nurmi, and Adlercreutz (2004) observed that mild acid hydrolysis separated acetyl and malonylic groups of isoflavone conjugates, releasing the 7‐*O*‐glycosides and aglycones from the food matrix. Walsh et al. (2003), on the other hand, found no difference in the concentration of isoflavones during in vitro digestion of soy bread in the mouth and stomach. These results may indicate that the stability of the isoflavones during simulated gastric digestion may vary with the food matrix and the pH prevailing in the process. Recovery of isoflavones was different for the tested samples: the value for sample IM was 1.6% and for sample II 6.1%. Inulin is not digested in the digestive tract due to the absence in gastric, pancreatic, and intestinal juice of enzymes hydrolyzing β‐(2 → 1) glycosidic bonds. Galazka, Klewicki, and Grzelak (2004) found that the most resistant to hydrolysis conducted under simulated gastric juice is crystalline inulin and inulin contained in a chicory flour. After 180 min of digestion, they observed 95% and 93% of the initial amount of inulin. This phenomenon may explain the highest recovery of isoflavones in the beverage with the addition of capsules containing inulin.

Specific isoflavones degraded to a different extent. 6″‐*O*‐malonylglycitin and 6″‐*O*‐malonylgenistin 1 were completely degraded in all test samples, while 6″‐*O*‐malonyldaidzin was more stable, and the recovery ranged from 3.6% (IMg) to 17.5% (IIg). It was similar in the case of glycosides. Recovery of glycitin and genistin was, respectively, 10.3% (IIg) and from 1% (IMg) to 2.6% (IIg), while that of daidzin ranged from 2.5% (IMg) to 9.0% (IIg). The consequence of greater degradation of genistein derivatives than daidzein derivatives was higher recovery of genistein than daidzein. Different levels of degradation and recovery of individual isoflavones could be due to the different chemical structure of the individual compounds. The presence of the hydroxyl group at C‐5 in genistein makes it more hydrophilic than daidzein, and therefore more prone to combine with a molecule of water. Hydroxyl groups, the specific position in the ring structures and the nature of substitutions may affect the biological properties of isoflavones.

After basification of digestive fluid (the second step of digestion), a twofold increase in the content of total isoflavones in the beverages containing microcapsules with maltodextrin, compared to the first step of digestion, was observed. For beverages fortified with inulin microcapsules during the second step of digestion, a decrease of isoflavones was observed. However, recovery of isoflavones for the tested samples IMi and IIi was comparable and was 3.3% and 3.7%, respectively. The increase in the content of isoflavones in the IMi sample may be caused by release of the isoflavones from the interior of the maltodextrin shell. Loss of the compounds in beverage IIi may have been associated with the degradation of free isoflavones on the surface of the microcapsule. Seok et al. (2003) found that in the conditions of the digestive tract, contents of isoflavones increase rapidly up to about 85%.

Similarly to the step of the stomach, at the colorectal stage, individual isoflavones showed different stability. During intestinal digestion, glycitin, genistein, and daidzein in the samples were completely degraded. Recovery of isoflavones varied depending on the nature of the sample. Glycoside and the malonylic derivative of daidzein were characterized by the highest stability during digestion. Recovery of daidzin ranged from 7.3% (IMi) to 8.7% (IIi), and that of 6″‐*O*‐malonyldaidzin was 6.9% (IMi, IIi). Recovery of genistin and 6″‐*O*‐malonylgenistin was, respectively, 2.7% and 3.9% in sample IMi and 2.8% and 5.1% in sample IIi.

In the conditions of simulated in vitro digestion in the membranes, only the glycosidic forms, that is, genistin and daidzin, were identified. Recovery of isoflavones was greater in the beverage sample with a maltodextrin (IMm), while it was smaller in the beverages fortified with the spray dried extract with inulin (IIm). The results may suggest that the process of microencapsulation increased bioavailability of the isoflavones in the gastrointestinal tract. Isoflavones were gradually released during subsequent stages of simulated in vitro digestion.

The efficiency of absorption of polyphenolic compounds from the gastrointestinal tract is affected by many factors, including hydrophilicity of the compound, the molecular weight, the environmental pH, the polymerization degree, and type of sugar molecule (Murota & Terao, 2003). Some flavonoid aglycones are hydrophobic and can be transported across biological membranes by passive diffusion. The combination of sugar with the aglycone in the form of a glycoside changes the nature of the compound to more hydrophilic, reducing the possibility of diffusion and the bioavailability of the compound. In the case of isoflavones, literature data do not indicate clearly which forms are better absorbed, and according to most authors glycosidic isoflavones have higher bioavailability, compared to aglycones. Walsh et al. (2003) observed higher bioavailability of glycosides than aglycones in the aqueous fraction of soy bread after in vitro digestion. Similar results were obtained by Manach, Williamson, Morand, Scalbert, and Rémésy (2005) and Setchell et al. (2002). In the studies of Rodríguez‐Roque et al. (2013), glycosides and aglycones showed bioavailability of 42% and 17%, suggesting that the glycosides contained in soy milk may be more available for absorption than aglycones after in vitro digestion. However, Izumi et al. (2000) described higher bioavailability of aglycones.

### 3.8 Antioxidant capacity of isotonic drinks

The antioxidant activity of beverages before and after digestion was measured by ABTS^+^ radical scavenging. Test results are given in Table 4. The antioxidant activity against ABTS^+^ in beverages before digestion was higher in samples fortified with the extract spray dried with maltodextrin (570.843 nmol TE/ml), and lower in the samples spray dried with inulin (320.097 nmol TE/ml). After digestion with liquid gastric and intestinal fluid, antioxidant activity against ABTS^+^ was reduced in all tested samples. The highest antioxidant activity, after simulated gastric digestion, was shown by isotonic drink with extract spray dried with maltodextrin (300.987 nmol TE/ml) and the lowest was shown by the beverage enriched in extract with inulin (100.011 nmol TE/ml). After the step of simulated intestinal digestion, samples with slightly antioxidant activity were obtained. In the IMi samples, a sixfold decrease in antioxidant activity (90.543 nmol TE/ml) was noted, compared to the sample before digestion, and in the IIi sample fourfold (80.942 nmol TE/ml). In the dialysate from the membranes, the activity was in the range from 30.965 nmol TE/ml (IIm) to 40.124 nmol TE/ml (IMm). Examining the relationship between the digestion step and the levels of phenolics and antioxidant activity, a positive correlation of samples was noted.

Gumienna, Goderska, and Czarnecki (2009), in their research on the digestion of white beans, observed a twofold decrease in antioxidant activity against ABTS^+^ radicals during stomach digestion and a fourfold increase in the intestinal digestion. Gawlik‐Dziki (2007), investigating in vitro digestion of wheat breads, noted that the ability to chelate iron after each digestion step was reduced and was correlated with the content of phenolic acids in the test samples. The ability to chelate Fe (II) was highest after the first stage of digestion and decreased with the progress of the digestion process.

### 3.9 Effect of microencapsulation and storage on content of isoflavones in isotonic drinks

Changes in the content of isoflavones during the storage of isotonic drinks (12 weeks, temperature 4°C and 30°C) are shown in Table 5. In beverages with capsules with insulin (II) and maltodextrin (IM) during storage, the content of individual isoflavones fluctuated. In beverage II, after 12 weeks, conversion of malonyl derivatives to the corresponding β‐glycosides (increase in the content of daidzein and glycine) and conversion of β‐glycosidic forms (genistein) to free aglycones was observed. As a result of these transformations, the content of genistein in beverages increased 300‐fold. It was different with IM beverages, because eventually, after 12 weeks of storage at 4 °C, the content of all isoflavones decreased.

**Table 5:**
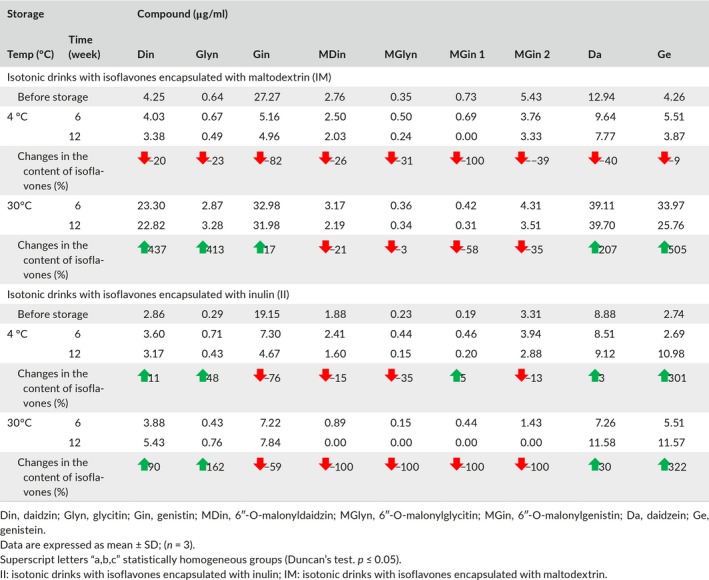
Content of isoflavones (μg/ml) indicated by HPLC in IM and II beverages before and after 6 and 12 weeks of storage at 4°C and 30°C

Isoflavones, as some authors state, undergo many changes under the influence of external factors and processes to which soybeans and soy products are subjected. For example, according to Kuo, Cheng, Wu, Huang, and Lee (2006) and Chun et al. (2007) fermentation removes the glucosidic group to release the aglycone. Extraction of soy isoflavones with boiling water to produce soy milk results in hydrolysis of the malonyl group, resulting in simple β‐glucosides (Barnes, Kirk, & Coward, 1994).

At 30°C in the beverage with maltodextrin capsules, the isoflavones gradually increased. Most likely maltodextrin capsules degraded and their contents went into solution. It can be concluded that the coatings of maltodextrin are not very stable at high temperatures, as was also mentioned in the works by Bae and Lee (2008) and Drusch, Serfert, Heuvel, and Schwartz (2006). Changes that occur in the structure of maltodextrin may cause interference in the compatibility between the carrier and the active substance. Guadarrama‐Lezama et al. (2014) evaluated the effect of storage temperature and water activity on the degradation of carotenoids contained in microcapsules (maltodextrin: arabic gum 1: 1) of chili extracts. The degradation of carotenoids was lower in microcapsules stored at 25°C than those stored at 35 or 40°C. The morphology of the microcapsules was changed with the water activity (*a*_w_) > 0.6. Water activity above 0.6 caused swelling of the polysaccharide matrix, which resulted in dissolution of the wall material and faster degradation of carotenoids.

In samples containing inulin capsules, the content of isoflavones changed slightly during the 12‐week storage at 30°C, which may indicate effective protection of these compounds in inulin capsules. Only malonyl derivatives were completely degraded. In studies of Glibowski and Bukowska (2011) it was found that in products with pH > 5, heated to 100°C, inulin was not degraded. Inulin may, however, have limited application in products with pH ≤ 4, heated to 60°C (Glibowski & Bukowska, 2011).

## 4 CONCLUSION

In the organoleptic assessment, isotonic drinks fortified with extract of microencapsulated isoflavones were rated higher than the drink with pure extract of isoflavones. With encapsulation, the taste, smell, and color of isoflavones became more acceptable to consumers. The study shows that it is preferable to prepare isotonic drinks with extracts spray dried with inulin. A better surface structure of the capsules and higher efficiency of microencapsulation was obtained using inulin as a carrier. Beverages enriched with inulin microcapsules were also characterized by better stability during storage. The highest recovery of isoflavones was observed for glycosidic and malonylic derivatives of daidzein, and bioavailability in the simulated gastrointestinal tract showed only glycosidic derivatives (daidzin, genistin). The results may suggest that the process of microencapsulation increased bioavailability of the isoflavones in the gastrointestinal tract.

Microencapsulated of isoflavones from soybeans can increase the health benefits of conventional beverages available on the market.

## CONFLICT OF INTEREST

The authors declare that there are no conflict of interests.
