# Supplementary material for: Effect of microencapsulation on concentration of isoflavones during simulated in vitro digestion of isotonic drink
Source: Food Sci Nutr. 2019 Jan 30;7(2):805–16. doi: 10.1002/fsn3.929 (PMC6392822; doi:10.1002/fsn3.929)
Supplement: Supplementary file 1 [file FSN3-7-805-s001.docx]

**Effect of the microencapsulation on the concentration of isoflavones during a simulated in vitro digestion of isotonic drink**

Dorota Wyspiańska, Alicja Z. Kucharska*, Anna Sokół-Łętowska, Joanna Kolniak-Ostek

Department of Fruit, Vegetable and Plant Nutraceutical Technology,

Wrocław University of Environmental and Life Sciences, Chełmońskiego 37, 51-630 Wrocław, Poland

***** Correspondence: [alicja.kucharska@upwr.edu.pl](mailto:alicja.kucharska@upwr.edu.pl) (AZ. Kucharska)

1

6

4

3

2

9

7

8

5

10

Gin d1

Gin d2

Fig. S1. UPLC-DAD chromatogram (280 nm) of preparation of soy isoflavones (Gin d 1, genistin derivative 1; Gin d 2, genistin derivative 2). The peak number corresponds to the number in Tables 3.

5

1

7

6

4

3

2

1

8

9

2

3

4

5

8

7

6

Gin d1

Gin d2

**B**

**A**

Fig. S2. ESI-MS chromatogram of preparation of soy isoflavones before (A) and after (B) fragmentation (Gin d1, genistin derivative 1; Gin d2, genistin derivative 2). The peak number corresponds to the number in Tables 3.
